# Mosquito Feeding Preference, Infectivity Rates, and Knockdown Resistance Within the Wild Population of *Anopheles arabiensis* in Jabi Tehnan District, Northwest Ethiopia

**DOI:** 10.3390/tropicalmed10100299

**Published:** 2025-10-21

**Authors:** Alemnesh Hailemariam Bedasso, Sisay Dugassa, Jimma Dinsa Deressa, Geremew Tasew Guma, Getachew Tolera Eticha, Mesay Hailu Dangisso, Eliningaya J. Kweka, Habte Tekie

**Affiliations:** 1 Ethiopian Public Health Institute, Addis Ababa P.O. Box 1242, Ethiopia; geremewtasew@gmail.com (G.T.G.); getachewtollera@gmail.com (G.T.E.); mesdang216@gmail.com (M.H.D.); 2 Department of Zoological Sciences, College of Natural and Computational Sciences, Addis Ababa University, Addis Ababa P.O. Box 1176, Ethiopia; habte_tm@yahoo.com; 3 Aklilu Lemma Institute of Pathobiology, Addis Ababa University, Addis Ababa P.O. Box 1176, Ethiopia; 4 Malaria and Neglected Tropical Diseases Research Division, Armauer Hansen Research Institute, Addis Ababa P.O. Box 1005, Ethiopia; jimmadinsa2@gmail.com; 5 Department of Medical Parasitology and Entomology, School of Medicine, Catholic University of Health and Allied Sciences—CUHAS, Mwanza P.O. Box 1464, Tanzania; 6 Pesticides Bioefficacy Section, Tanzania Plant Health and Pesticides Authority, Arusha P.O. Box 3024, Tanzania; 7 Bugando Medical Centre, Mwanza P.O. Box 1370, Tanzania

**Keywords:** *Anopheles*, blood meal, host-seeking, resting, malaria, Jabi Tehnan

## Abstract

**Background:** In recent decades, malaria vector species distribution and insecticide resistance have taken new colonization steps across Africa. Understanding the malaria vector insecticide resistance status, blood meal source, and species composition is of paramount importance in designing evidence-based vector control strategies. This study assessed the blood meal sources, sporozoite (infectivity) rate, and knockdown resistance allele’s frequency in female *Anopheles arabiensis* in chosen villages of Jabi Tehnan District, Northwest Ethiopia. **Methods:** The host-seeking and resting *Anopheles gambiae* s.l. were collected using human landing catches (HLCs), CDC light traps (CDC-LTs), pyrethrum spray catches (PSCs), and pit shelters (PSs) both indoors and outdoors. The analysis of both blood meal sources and circumsporozoite proteins was performed using enzyme-linked immunosorbent assay (ELISA). The detection of knockdown resistance gene mutations and species identification were conducted using a polymerase chain reaction (PCR). **Results:** A total of 5098 female *Anopheles gambiae* s.l. were collected. Of these, 1690 (33.2%) were collected from HLCs, 1423 (27.9%) from CDC light traps, 1635 (32.0%) from PSCs, and only 350 (6.9%) from pit shelters (PSs). Of these, 57.2% (n = 2915) female *Anopheles* mosquitoes were collected indoors using CDC light traps (CDC-LTs), human landing catches (HLCs), and pyrethrum spray catches (PSCs), while 38.2% (n = 2183) were collected outdoors using human landing collection (HLC), CDC light traps (CDC-LTs), and artificial pit shelters (PSs). Molecular identification to the species level showed that among the 530 *An. gambiae* s.l. samples analyzed using PCR, 96.03% (509) were *An. arabiensis*, and 3.97% (21) were unidentified species. The biting peak was found to be from 22:00 to 00:00 h for *An. arabiensis*. However, their activity decreased sharply after 23:00 to 00:00 h. The distribution of knockdown resistance genes in the tested specimens of *An. arabiensis* consisted of 1.4% (n = 3) heterozygous resistant (RS), 17.9% (n = 38) homozygous resistant (RR), and 80.7% (n = 171) homozygous susceptible (SS) genotypes. A higher proportion of *Anopheles* mosquitoes analyzed for blood meal analysis had a human blood meal origin at 13.1% (n = 47), followed by bovine at 8.9% (n = 32) and mixed at 5.8% (n = 21). **Conclusions:** The dominant malaria vector species was *Anopheles arabiensis* in the study area with a higher human blood meal origin. The Kdr gene was confirmed in the tested *An. arabiensis*, indicating that an alternative insecticide class should be used in the study area.

## 1. Introduction

*Anopheles arabiensis* and *An. gambiae* s.s. are the major malaria vectors in the Afrotropical region, which can survive in a range of altitudes, temperatures, and humidity conditions [1,2] and are sympatric in many regions [2,3], while those in the *Anopheles funestus* group serve as secondary vectors [4]. The mosquito species that breed in freshwater habitats are sympatrically distributed with other species of the *An. gambiae* complex [2]. *An. gambiae* s.l. is dominant in the humid parts of Africa with freshwater, while *An. arabiensis* is more widespread in the dry savannah habitats of Africa [5]. Contrarily to the findings of ecological studies that have shown that *arabiensis* and *An. gambiae* breed in clean water, recently, they have been found to survive in polluted habitats [6,7,8]. The accurate identification of malaria vector species is critical for designing and deploying efficacious tools for vector control [9]. The morphological keys developed by Gillies and Coetzee [1,10] and rigorous molecular analysis via a polymerase chain reaction (PCR) are used for the identification of sibling species in the *An. gambiae* and *An. funestus* complexes [11,12] and are important for effective malaria vector control programs [12,13].

Apart from species identification and host preference, it is important to understand the vectorial capacity and malaria transmission dynamics of *Anopheles* species [3,14]. *Anopheles* mosquitoes have a wide range of preferred hosts, including humans and animals such as cattle, sheep, horses, pigs, dogs, cats, birds, and reptiles, as well as other vertebrates and reptiles [15,16]. Anthropophilic *Anopheles* mosquitoes usually feed on humans, whereas zoophagic *Anophelines* feed mainly on bovines [17,18]. However, others are opportunistic, feeding on both humans and other animals depending on which host species is available [19].

The blood-feeding behavior of host-seeking female *Anopheles* mosquitoes is one of the drivers of the transmission of *Plasmodium* parasites in human populations during endemic malaria transmission seasons [20]. The human blood index (HBI), expressed as the proportion of mosquito blood meals that are of human origin, is an important indicator of malaria transmission and is used to estimate the human biting rate to measure the vectorial capacity and intensity of malaria transmission [21].

The sporozoite infectivity rates of mosquitoes are utilized to better understand malaria transmission intensity, vector species importance, and intervention impact [22]. The *Plasmodium falciparum* circumsporozoite protein for *P. vivax*-210 or *P. vivax*-247 is also used [23]. These phenomena are of paramount importance in malaria vector intervention evaluation and impact assessment [9].

*Anopheles arabiensis* is a main vector species of malaria in Ethiopia which has developed resistance to multiple insecticides. This is probably a result of the long-term use of indoor residual spraying (IRS) and insecticide-treated bed nets (ITNs) and their use in agriculture [24,25]. The increased multiple insecticide resistance of the main malaria vectors has raised concerns regarding insecticide-based malaria vector control interventions [26]. Insecticide resistance has been detected using molecular assays, which give early notice of resistance emergence and the underlying mechanism [3,27,28,29]. This has improved the precision of the insecticides chosen [30,31].

The relationship between the knockdown resistance (kdr) mutation, blood meal sources, and *Plasmodium* infection in Ethiopia is not well understood. These parameters are of paramount importance in designing appropriate approaches for effective vector control tools and programs. In Ethiopia, previous studies have identified insecticide resistance to *An. arabiensis* in multiple insecticide classes [32]. However, data are scarce in Jabi Tehnan District. Insecticide-based methods for controlling malaria vectors have proven to be successful in many parts of Africa [33]. Yet, because of the rise in insecticide resistance among the main malaria vectors, the fear of insecticide-based malaria vector control tools failing has grown. One of the mechanisms of the development of resistance is the knockdown resistance (kdr) mutation, which transforms Leucine (TTA) into Phenylalanine (TTT) at position 1014 of the voltage-gated sodium channel (VGSC) [32,34].

This study investigated malaria vector species diversity, blood meal source, and *Plasmodium* circumsporozoite rates to address challenges facing African and Ethiopian malaria control initiatives, such as insecticide resistance [35]. Establishing effective techniques for managing insecticide resistance and maintaining the effectiveness of chemical-based vector control tools can be aided by a well-coordinated national database on knockdown resistance, malaria vector species diversity and dynamics, blood meal sources, and *Plasmodium* circumsporozoite rates. These must be understood to create effective vector control strategies and procedures and to manage residual malaria transmission with particular care.

This study provides new insights compared with previous Ethiopian studies because it focuses on Jabi Tehnan District, a location with unique ecological, epidemiological, and intervention-related contexts that have not been extensively characterized. The district’s agricultural practices, climate variability, and rapid changes in land use create distinct vector habitats and transmission dynamics that differ from those previously documented in other regions of Ethiopia. Furthermore, the study period coincides with the intensified implementation of malaria control interventions, which included the expanded coverage of indoor residual spray (IRS), long-lasting insecticidal nets (LLINs), and new policy plans on the diagnostics and availability of first-line anti-malaria treatments. Conducting this research during a time of significant environmental change and evolving intervention strategies allows for the identification of emerging entomological and epidemiological patterns that may not have been captured in earlier studies, thereby filling a critical knowledge gap for localized malaria elimination efforts.

## 2. Materials and Methods

### 2.1. Description of Study Area and Study Design

**Figure 1:**
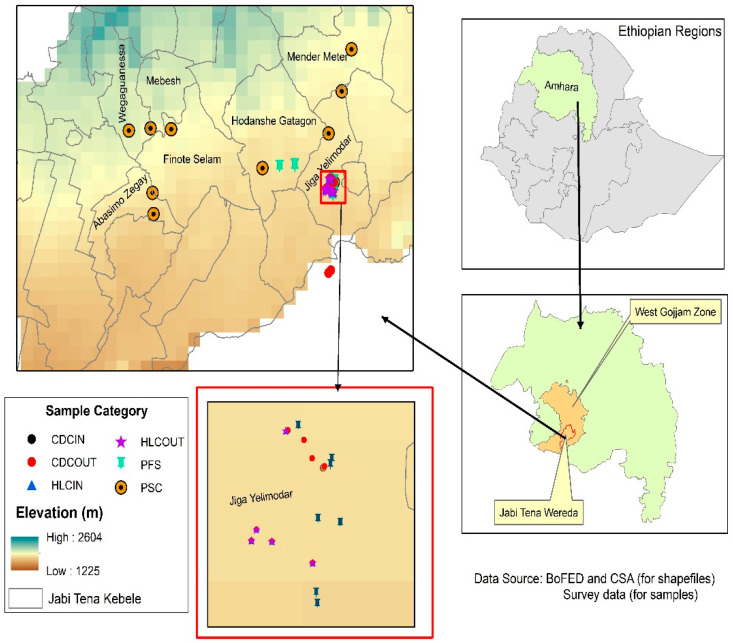
Map showing Jabi Tehnan District, West Gojam Zone, Amhara Region, Ethiopia, where this study was conducted.

This study was conducted in Jabi Tehnan District, a malaria-endemic area in West Gojjam Zone, Northwest Ethiopia, that is located at a distance of 387 km from Addis Ababa and 176 km southwest of Bahir Dar City (Figure 1). This area lies between 10°24′36″ and 10°55′48″ N latitude and 37°4′12″ and 37°30′36″ E longitude. This study was conducted at an altitude ranging from 1345 to 2572 masl [36]. A considerable part of the area lies in ranges closer to 2000 masl where malaria is endemic. The area receives a rainfall of about 1250 mm per annum, and the mean minimum and maximum temperatures are 14 °C and 32 °C, respectively. The rainfall distribution in the area is unimodal, and the rainy season lasts from June to mid-September [37]. The district’s total population is 225,769, of which 112,341 are males, and 113,428 are females [36,37,38].

A total of five villages were selected in Jabi Tehnan District for this study: Jiga Yelimodar (37°21′36″ E; 10°40′48″ N; altitude: 1833 m), Hodanshe Gatagon (37°19′12″ E; 10°42′00″ N; altitude: 1866 m), Abasimo Zegay (37°13′12″ E; 10°39′00″ N; altitude: 1806 m), Mender Meter (37°21′36″ E; 10°45′00″ N; altitude: 1947 m), and Mebesh (37°14′24″ E; 10°43′48″ N; altitude: 2037 m). These villages were purposively selected based on documented malaria endemicity [36,37], the representation of a range of altitudes (1806–2037 m), and ecological settings to capture variation in malaria transmission, accessibility for field surveys and logistical feasibility, and their relevance to ongoing malaria control interventions in the district.

Households were selected based on several ecological, structural, and social criteria. Proximity to mosquito breeding sites was a key factor, with priority given to houses located near known or potential *Anopheles* larval habitats such as streams, ponds, irrigation canals, and rice fields to maximize mosquito collection efficiency. Housing structures of varying construction types (e.g., thatched versus corrugated iron roofs, mud versus cement walls, and presence of eaves) were included to assess the influence of building characteristics on mosquito entry and resting behavior. Socio-environmental factors, including household practices and environmental features such as livestock presence, water storage practices, and vegetation cover, were also considered given their potential effect on mosquito density and human–vector contact. To avoid spatial clustering and ensure ecological representativeness, sentinel houses were distributed across each village to capture different microhabitats. All community members who provided consent and were accessible to the research team were voluntarily recruited for this study.

### 2.2. Survey of Vertebrate Hosts

Data on the population of vertebrate hosts residing in the study sites were provided [39]. Similar information was gathered from this study for potential hosts, including bovines, goats, dogs, and chickens [40].

### 2.3. Adult Mosquito Sampling Methods and Processing Procedures

The collection of adult mosquitoes was conducted from September 2016 to August 2017, using four WHO-recommended sampling techniques: pyrethrum spray catches (PSCs), human landing catches (HLCs), Centers for Disease Control (CDC) light traps, and pit shelters (PSs) [41]. Generally, these sampling methods were used for the collection of adult mosquitoes indoors and outdoors.

Five houses were selected in each sentinel village to conduct human landing catches (HLCs) and ten houses each for collection using pyrethrum spray catches (PSCs) and CDC light traps (CDC-LTs). Houses were chosen via simple random sampling from a list of all occupied households in the village after stratification by proximity to known larval habitats (within 500 m vs. >500 m), ensuring the representation of both near- and far-from-breeding-site households. The same houses were used throughout the study period unless a household became unavailable, in which case a pre-randomized replacement was enrolled.

The number of houses per method was determined by balancing spatial coverage, ethical considerations, logistical feasibility, and statistical power. HLCs were conducted at five houses per village to maximize the coverage of biting activity across settlements while minimizing collector workload and limiting exposure risk (see Ethics). PSCs and CDC-LTs were conducted at ten houses per village to provide robust estimates of indoor resting densities and trap-based abundance, complementing HLC data. Collections using different methods were conducted concurrently where possible to enable direct comparisons. The chosen sampling scheme (5 houses for HLCs and 10 houses for PSCs and CDC-LTs per village) lies within the range commonly applied in entomological surveys in Ethiopia and elsewhere (typically 3–6 houses per method per village) while providing sufficient replication across sites to ensure representative estimates of mosquito density and behavior.

### 2.4. Human Landing Catches (HLCs)

In each study site, five houses were randomly selected as sentinel houses for human landing catches. In each of the five houses, indoor and outdoor mosquito collections were carried out from 6:00 pm to 6:00 am for four successive nights. HLCs were carried out monthly. In each village, mosquitoes were collected from four houses, each separated by approximately 100 m. The weather data were recorded in each house that received IRS [42].

Human landing catches (HLCs) were conducted by trained collectors who were provided with mefloquine chemoprophylaxis, rather than by untrained village volunteers. This approach ensured both the safety of participants and the reliability and consistency of mosquito collection data. HLCs were conducted by an adult man exposing his lower limbs and collecting landing mosquitoes using an aspirator [41]. Each mosquito collected stayed in a station for 45 min, and there was a 15 min break for resting. To obtain hourly biting densities, the catches for each hour were divided by 0.75 [43]. In each house, a group of 4 data collectors was deployed. Once the team members arrived at the collection household, they were divided into two groups of two collectors each. One group was set to trap indoors, while the other group was outdoors. Outdoor mosquito sampling groups sat between 8 and 10 m apart from each other in all sentinel houses selected for indoor mosquito collection. There were two collection shifts: one team of collectors worked from 18:00 to 00:00 h, followed by the second team working from 01:00 to 06:00 h.

In each hour, volunteers rotated between indoors and outdoors to avoid positional bias. A full dose of mefloquine was provided as chemoprophylaxis to the volunteers who participated in human landing collections. Daily follow-up was also performed on all volunteers for any malaria symptoms and prompt access to treatment with effective anti-malarial drugs if any of the collectors were screened and then found to have malaria parasites. Captured mosquitoes were placed in labeled paper cups at hourly intervals. Thus, a total of 24 labeled paper cups (12 labeled cups for indoor and 12 labeled cups for outdoor collection) were used for each night collection per house. At the end of each collection night, all paper cups with mosquitoes were brought to the field identification center where the identification of mosquitoes to the species level using taxonomic keys [1,10] and their physiological status, unfed (UF), fed (F), half-gravid (HG), or gravid (G), was performed [41].

### 2.5. Pyrethrum Spray Catches

In each sentinel village, ten houses were randomly chosen for pyrethrum spray catches (PSCs). Mosquitoes were sampled from 6:00 a.m. to 7:00 a.m. from each house for two consecutive nights in all ten selected houses. Before PSCs, all the things and food in the room were covered with white sheets, and all animals were removed from the room where the collection took place. A commercially available pyrethroid-based aerosol (Baygon aerosol, SC. Johnson & Son Inc., Racine, WI, USA) was sprayed in the entire space of the room, and the house was then closed for 15 min after spraying. After fifteen minutes, all the knocked-down mosquitoes on the white sheets were collected carefully with forceps and placed in paper cups [41]. All *Anopheles* mosquitoes collected on the white cotton sheet were identified to species using taxonomic keys, and the abdominal stage of each mosquito was also determined as unfed, freshly fed, half-gravid, or gravid [41].

### 2.6. CDC Light Traps (CDC-LTs)

CDC miniature light traps (Gladwick St., Rancho Dominguez, CA, USA, and J.W. Hock Ltd., Gainsville, FL, USA) were powered by a GS premium high-power (6N11-2D; 6v-11Ah/10Hr Japan storage battery Co., Ltd., Kyoto, Japan), rechargeable battery. The battery was set near an occupied bed at a height of 1.5 above the floor from 18:00 to 06:00 h to sample indoor host-searching mosquitoes [41]. For outdoor host-seeking mosquito sampling, a CDC light trap was also set in the vicinity (within 2 m) of sentinel houses from 18:00 to 06:00 h. Ten houses with paired traps indoors and outdoors in the same village were selected with varied vicinities to breeding. Mosquitoes were collected indoors and outdoors from 18:00 to 06:00 h from each house using CDC light traps. Mosquito collection bags were retrieved from traps in the morning between 06:00 h and 07:00 h. Mosquitoes were identified using morphological features [1,10] and classified based on abdominal status (blood-fed, empty (unfed), semi-gravid, and gravid) [41].

### 2.7. Pit Shelter Collections

Outdoor resting mosquitoes were collected monthly between 06:00 and 09:00 h from ten pit shelters made in selected house compounds. A rectangular pit was dug in the ground (1.5 m in depth, 1.2 m in length, and 1 m in width). Mosquitoes were sampled monthly using a mouth-suction aspirator according to the method stated in the WHO entomological manual in 1975 [41]. The captured mosquitoes were anesthetized using chloroform. The pit shelters were covered with an untreated net during collection to prevent mosquitoes from escaping [41].

### 2.8. Molecular Identification of Anopheles gambiae s.l.

DNA was extracted from the legs and wings of the identified female Anopheles mosquitoes using the DNeasy Blood and Tissue Kit using the manufacturer’s protocol (Qiagen^®^, Sigma Aldrich, St. Louis, MO, USA). The extracted DNA was stored at −20 °C until it was used for molecular analysis. Then, it was subjected to molecular identification using a species-specific polymerase chain reaction (PCR) assay following the methods of Scott and others [12], at the Molecular Biology Laboratory of Tropical and Infectious Diseases Research Centre (TIDRC), Sekoru, Jimma University. Briefly, PCR amplification was performed in a final reaction of 20 uL using a set of each primer with a 0.25 µM final concentration (UN: 5′-GTGTGCCCCTTCCTCGATGT-3′) and species-specific primers for *An. gambiae* s.l (GA: 5′-CTGGTTTGGTCGGCACGTTT-3′), *An. arabiensis* (AR: 5′-AAGTGTCCTTCTCCA TCCTA-3′), 7.5 µL nuclease-free water, and 1 µL template DNA. The PCR program was set for an initial step at 94 °C/10 min, 30 cycles of 94 °C/30 s, 50 °C/30 s, 72 °C/30 s, and final extension at 72 °C/5 min. Then, the amplicon was loaded on 2% agarose gel stained with 1% ethidium bromide, and a 100 bp reference DNA ladder was used. Finally, the band size of PCR products was compared to the *An. arabiensis*-susceptible colony strain used as a positive control for confirmation during visualization on a Bio-Rad-UV-gel documentation system.

### 2.9. Detection of Knockdown Resistance Gene Mutation

An analysis of the knockdown resistance gene (L1014F) mutation was carried out on 251 *An. arabiensis* (PCR-identified) samples as described in previous reports conducted in different areas with species identifications [44,45]. Briefly, genomic DNA extracted from individual mosquitoes was genotyped using allele-specific primers [44]. The amplicon was run on a 2% agarose gel and stained with 3 µL of 1% ethidium bromide. The resulting fragments (bands) were interpreted as 293 bp internal control, 195 bp resistant, and 137 bp susceptible/wild-type mosquitoes [13,44]. Susceptible *An. arabiensis* strains were taken from the Sekoru insectary colony of Jimma University, Tropical and Infectious Diseases Research Center, Ethiopia, and used as a control.

### 2.10. Analysis of Mosquito Blood Meal Source

The origins of the blood meals of all freshly fed mosquitoes and a subset of half-gravid Anopheles mosquitoes (*An. arabiensis*) were identified using ELISA. Adult mosquitoes were collected from a malaria-endemic village, and blood-fed specimens were analyzed following the method of Beier et al. [46].

Briefly, the abdomen of each freshly blood-fed mosquito was separated from the head–thorax and homogenized in phosphate-buffered saline (PBS) using a pestle in a 1.5 mL Eppendorf tube. The homogenate was diluted 1:50 with PBS, and 50 μL of the diluted sample was added to U-shaped microplate wells. After incubation at room temperature for 3 h, the homogenate was discarded, and the wells were washed twice with PBS–Tween 20. Subsequently, 50 μL of peroxidase-conjugated human antibodies and 50 μL of phosphatase-conjugated bovine antibodies were added to the respective wells. Plates were then covered and incubated for 1 h at room temperature, followed by three washes with PBS–Tween 20. Finally, 100 μL of ABTS peroxidase substrate solution was added, and absorbance was measured using an ELISA reader at 405–415 nm after 30 min. Positive controls consisted of cattle and human blood dried on Whatman paper, while negative controls included unfed *An. arabiensis* from an insectary colony at ALIPB.

### 2.11. Determination of Plasmodium Sporozoite Rates

The head and thorax of preserved *An. gambiae* s.l. mosquitoes were carefully separated from the abdomen and processed for the detection of the circumsporozoite protein for both *P. falciparum* and *P. vivax* using an enzyme-linked immunosorbent assay (CSP-ELISA) [47]). The wavelength absorbance was measured at 405 nm using a UV-assisted spectrophotometer ELISA reader (SpectraMax i3x (Syngene International Ltd., Baltimore, MD, USA)). The sporozoite rate was estimated as the number of mosquitoes with sporozoites divided by the number of females examined multiplied by 100 [48].

### 2.12. Data Analysis

The human blood index (HBI) and bovine blood index (BBI) were calculated as the ratio of blood-fed mosquitoes that had fed on humans and cattle, respectively, relative to the total number of mosquitoes tested. The mixed blood meal source was determined as the ratio of blood-fed mosquitoes that had fed on both human and cattle blood to the total tested represented as a percentage. The unknown blood meal source represented the proportion of blood-fed mosquitoes with unidentified blood origins, also expressed as a percentage [14]. The sporozoite rate (SR) was calculated as the number of mosquitoes positive for circumsporozoite protein (CSP) antigens divided by the total number examined, expressed as a percentage [48,49].

## 3. Results

### 3.1. Survey of Vertebrate Hosts in Study Site

**Figure 2:**
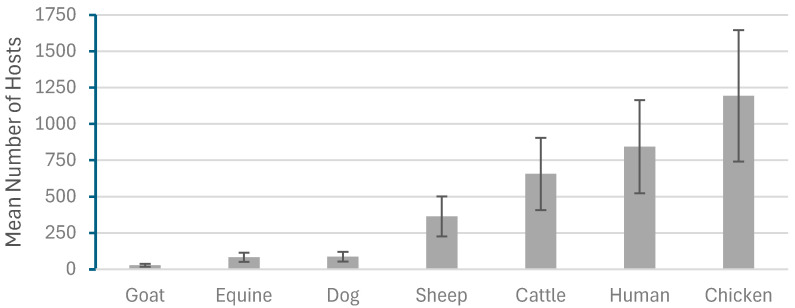
The host species population variation during data collection in the study area.

A total of 19,520 potential vertebrate hosts were recorded in the study villages (Table 1), including cattle, goats, sheep, dogs, chickens, equines, and humans, which varied statistically significantly (F = 23.74, df = 6, *p* > 0.001) (Figure 2).

### 3.2. Anopheles Mosquito Species Compositions and Abdominal Status Using Different Sampling Techniques

A total of 5098 female Anopheles mosquitoes belonging to five species were collected using four sampling techniques: CDC light traps (1423, 27.9%), human landing catches (HLCs) (1690, 33.2%), pyrethrum spray catches (PSCs) (1635, 32.1%), and pit shelters (PSs) (350, 6.9%). Of these mosquitoes, 57.2% (n = 2915) were sampled indoors using PSCs, HLCs, and CDC-LTs, while 42.8% (n = 2183) were sampled outdoors using HLCs, CDC-LTs, and PSs (Table 2).

The examination of abdominal status showed that the majority of mosquitoes were unfed (2792, 54.8%), followed by freshly fed (2126, 41.7%), gravid (147, 2.9%), and half-gravid (33, 0.6%). Species-specific analysis indicated that *An. arabiensis* was the most abundant (2982, 58.5% of the total), with a high proportion of unfed and freshly fed females, suggesting active host-seeking and blood-feeding behavior. Other species collected included *An. coustani* (774, 15.2%), An. cinereus (561, 11.0%), *An. pharoensis* (605, 11.9%), and *An. funestus* (176, 3.5%).

The distribution of abdominal status across sampling methods showed that HLCs captured the highest number of host-seeking (unfed) mosquitoes outdoors, while PSCs and CDC-LTs captured primarily indoor-resting freshly fed mosquitoes. Pit shelters mainly collected outdoor-resting mosquitoes with a mix of those of unfed and freshly fed status (Table 2). These patterns highlight the behavioral diversity of vector species and emphasize the importance of using multiple sampling methods to accurately assess malaria transmission potential.

### 3.3. Molecular Identification of Anopheles gambiae s.l. Mosquitoes

**Figure 3:**
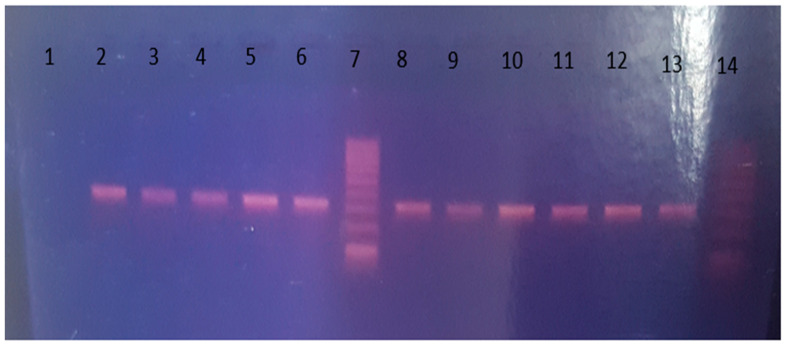
Results of PCR gel electrophoresis: lane 1—negative control; lanes 2–6 and 8–13—315 bp *Anopheles arabiensis.* Lanes 7 and 14 were 100 bp DNA ladders.

Among the 530 *An. gambiae* s.l. samples analyzed using species-specific PCR, 509 (96.03%) were *An. arabiensis*, while the remaining 21 (3.97%) of the samples were not amplified (unknown) (Figure 3).

### 3.4. Detection of Knockdown Resistance Gene Mutation

The presence of the L1014F (West African kdr) allelic mutation was investigated in 251 PCR-confirmed *An. arabiensis* mosquitoes, of which 84.5% (n = 212) were successfully amplified, and 15.5% (n = 39) were unamplified. The knockdown resistance gene distribution consisted of 1.4% (n = 3) heterozygous resistant (RS), 16.3% (n = 38) homozygous resistant (RR), and 68.13% (n = 171) homozygous susceptible (SS) genotypes, and 15.54% (n = 39) were undetected (NB) (Table 3).

### 3.5. Blood Meal Sources of An. arabiensis Mosquitoes

The human blood index (HBI) and bovine blood index (BBI) and the mixed-blood-fed and unknown blood meal sources of *An. arabiensis* are presented in Table 4. Out of 1475 freshly fed field-caught *An. arabiensis*, 360 (24.4%) mosquitoes were tested for single-host blood meals, mixed blood meals, and unknown blood meal sources. For blood meal analysis, the following vertebrate antisera were used: human and bovine. These were chosen based on the common domestic hosts present in the study villages, allowing for the determination of the host-feeding patterns of *Anopheles arabiensis*. Among the tested mosquitoes, 13.1% (n = 47) of blood meals were of human origin, 8.9% (n = 32) were bovine, and 5.8% (n = 21) were mixed (human and bovine), while the majority, 72.2% (n = 260), were from unidentified sources. Mosquitoes collected with pyrethrum spray catches (PSCs) exhibited a relatively higher HBI (17.7%, n = 23) and BBI (9.2%, n = 12) compared to those captured using human landing catches (HLCs), CDC light traps (CDC-LTs), and pit shelters (PSs).

### 3.6. Plasmodium Circumsporozoite Protein Detection in Anopheles Mosquitoes

A total of 500 *An. arabiensis* and 20 *An. pharoensis* mosquitoes were assessed for the presence of *Plasmodium falciparum* and *P. vivax* circumsporozoite protein (CSP). No mosquitoes tested positive for *P. falciparum* CSP; therefore the entomological inoculation rate (EIR) was not calculated.

## 4. Discussion

The findings of this study showed that understanding blood meal sources, the detection of knockdown genes, and species composition is of paramount importance for the effective design of the malaria vector control programs and tools to be used. The understanding of mosquito host choice behavior during blood meal analysis was found to be crucial in providing information on how vectors transmit pathogens between vertebrates. In natural settings, mosquitoes exhibit varied feeding succession due to host availability and attractiveness [50]. The feeding patterns and host associations of mosquitoes are influenced by both environmental and biological factors [51]. Female mosquitoes ingest vertebrate blood to produce eggs and then transmit pathogens during blood feeding, and those that feed on multiple hosts like humans and other hosts accelerate malaria transmission by shortening the incubation period [52]. In Ethiopia, the primary vector feeding on human blood is *An. arabiensis*, with great implications for malaria transmission, followed by *An. stephensi*, and these vectors must be tackled to control and eliminate them [53]. In female *An. arabiensis*, after blood feeding, their abdomens become engorged, which affects their flight capacity, host-seeking behavior, and patterns, while half-gravid mosquitoes prefer resting sites like suitable water resources to lay eggs [54]. In this study, we performed laboratory investigations on 500 *An. arabiensis* and 20 *An. pharoensis* mosquitoes to determine the mosquito infectivity rate, which refers to the proportion of those carrying infectious plasmodium sporozoites, using the sandwiched enzyme linked immunosorbent assay method. None of the analyzed mosquitoes were positive for either *Plasmodium falciparum* or *Plasmodium vivax* infections, considering the limitations of the method, and due to this, we were unable to determine the entomological inoculation rate (EIR). However, this may not represent the real situation of the study area, and therefore large-scale surveillance is recommended as vector density and malaria parasite incidence are resurging.

This study provides a comprehensive assessment of Anopheles mosquito species composition, abdominal status, host-feeding behavior, and infection potential in Jabi Tehnan District, Northwest Ethiopia. A total of 5098 female mosquitoes were collected, with *An. arabiensis* being the most abundant (2982, 58.5%), followed by *An. coustani* (15.2%), *An. pharoensis* (11.9%), *An. cinereus* (11.0%), and *An. funestus* (3.5%). An analysis of abdominal status revealed that most mosquitoes were unfed (54.8%) or freshly fed (41.7%), indicating active host-seeking and recent human–vector contact, while half-gravid and gravid females were relatively sparse, suggesting pre-oviposition behavior.

Indoor versus outdoor collections showed that 57.2% of mosquitoes were captured indoors using PSCs, CDC light traps, and HLCs, while 42.8% were collected outdoors using HLCs, CDC-LTs, and pit shelters. This indicates the presence of both endophagic/endophilic and exophagic/exophilic vectors. These patterns highlight the importance of integrated vector control strategies that target multiple ecological niches and both indoor and outdoor biting populations.

The laboratory testing of 500 *An. arabiensis* and 20 *An. pharoensis* mosquitoes revealed no CSP-positive mosquitoes; hence the calculation of the entomological inoculation rate (EIR) was not possible. This absence may reflect seasonality, limited sample size, or assay sensitivity, rather than a true lack of malaria transmission. Coupled with the high abundance of host-seeking and recently fed mosquitoes, these findings suggest that transmission potential remains in the area. Blood meal behavior further demonstrates that vectors feeding on multiple hosts, including humans, can accelerate malaria transmission by shortening the parasite incubation period.

The 17.9% frequency of resistance alleles suggests moderate insecticide resistance in the local vector population, comparable to other mid-altitude Ethiopian sites (10–25%) and lower than in some East African regions (>40%) [55]. A 15.5% PCR amplification failure rate is unlikely to significantly bias this estimate if the failures were randomly distributed, but ongoing monitoring is essential to detect changes that may compromise LLIN and IRS effectiveness.

The findings of this study highlighted the areas to focus on to strengthen indoor interventions such as LLINs and IRS. Scaling up blood meal and sporozoite monitoring will help guide timely and evidence-based interventions. Overall, this study underscores the need for integrated, evidence-based vector control strategies that account for species diversity, feeding behavior, abdominal status, insecticide resistance, and both indoor and outdoor transmission dynamics to effectively reduce malaria risk in the district.

This study identified five *Anopheline* species morphologically. These are *An. arabiensis*, *An. funestus*, *An. pharoensis*, *An. coustani*, and *Anopheles cinereus*, which were found to be similar to those in earlier studies in other parts of the region [56,57]. Among these, the *An. arabiensis* species was confirmed by a molecular test to be able to be used to determine the frequency of insecticide resistance. The large-scale use of insecticide-treated nets (ITNs) and indoor residual spraying is associated with an increased number of cases, and management led to a remarkable reduction in malaria cases and vector control up to early 2019 in Ethiopia [58]. In most African countries, following the massive use of insecticides, insecticide-resistant vectors are most commonly seen in malaria-endemic settings [59]. According to a study by Karunaratne and others, the metabolism and longevity of insecticide-resistant *An. gambiae* are lower than those of the susceptible strain, and the resistant stain showed a higher level of oxygen reactive species (ROS), which are key factors in determining oxidative stress. The major effects of insecticide resistance in mosquitoes are metabolic changes, altered target sites, and cuticular and behavioral resistance [60]. Overall, mosquitoes that develop the capacity to cope with oxidative stress are likely to live longer. According to an experimental investigation study by Oliver and Brooke, evaluating the effects of oxidative stress on the longevity of both *An. arabiensis* and *An. funestus* bearing, respectively, kdr and cytochrome P450 mechanisms demonstrates that these species live longer and that cytochrome P450 activity seems to be more protective against oxidative stress [61]. In our study, among the *An. arabiensis* species confirmed by a molecular test, 251 genomic isolates were tested for kdr, and the frequency was determined, as indicated in Table 3. Thus, we detected L1014F (West African kdr) allelic variant point mutations in 17.9% of the *An. arabiensis* mosquitoes genotyped, and over 80% were the susceptible strain. This study provides valuable insights into and information about the mosquito infectivity rate, blood-feeding patterns, mosquito density, and allelic frequency of insecticide resistance in the study setting.

The reported 17.9% frequency of the resistance allele in the study area indicates a moderate level of insecticide resistance within the local Anopheles population. This value is comparable to previous findings in mid-altitude Ethiopian sites, where resistance allele frequencies ranged from 10% to 25% [62,63], and is lower than the frequencies reported in certain high-transmission areas of East Africa, such as western Kenya and Tanzania, where frequencies often exceed 40–50% [64,65]. The observed allele frequency suggests that while resistance is present, it has not yet reached fixation in the local population. Continuous monitoring is warranted, as rising resistance levels could compromise the effectiveness of indoor-based interventions such as LLINs and IRS. Moreover, these findings emphasize the need for integrated vector management strategies, including the rotation of insecticides and complementary control measures, to mitigate further selection pressure.

This study found that 15.5% of all specimens failed to be amplified to the species level during molecular analysis, which could be due to factors such as degraded DNA, insufficient template quantity, or suboptimal PCR conditions. Also, the reported presence of the invasive mosquito species *An. stephensi* might be the cause as it is already widely reported in Ethiopia [66,67,68,69]. These “not amplified” samples may introduce potential bias in estimating resistance allele frequencies if their distribution differs from that of successfully amplified samples. For instance, if non-amplified mosquitoes disproportionately carried the resistance allele or belonged to a specific species, the reported 17.9% allele frequency could underestimate or overestimate the true prevalence. However, assuming the failures occurred randomly across species and collection sites, the impact on overall allele frequency estimates is likely minimal. Nevertheless, reporting the amplification failure rate and considering it in data interpretation are important to provide an accurate assessment of resistance in the local vector population.

Overall, we recommend that combination efforts targeting vector bionomics and parasite clearance and the large-scale molecular surveillance of insecticide resistance are very important for the management of vector and malaria parasite incidence, which is resurging in Ethiopia.

## 5. Conclusions

This study gives valuable insights into malaria transmission dynamics and vector control strategies. Blood-feeding patterns influence malaria transmission dynamics as *An. arabiensis* is the primary vector feeding on human blood in Ethiopia. Thus, insecticide-resistant vectors are common in malaria-endemic regions, and large-scale molecular surveillance is recommended for accurate vector density, insecticide resistance, and malaria incidence assessment.

To strengthen future blood meal analyses and reduce the proportion of unidentified samples, studies should expand the panel of tested hosts to include locally relevant species, adopt molecular approaches such as cytochrome b or 16S rRNA sequencing for higher sensitivity, and improve field preservation protocols. These efforts, complemented by contextual household or ecological data on host availability, would provide a more accurate understanding of vector feeding behavior.

A substantial proportion of blood meals in this study were classified as “unknown”, which limits our ability to fully characterized host-feeding patterns. Several factors may have contributed to this. First, the antiserum panel used (human, bovine, goat, chicken) did not cover all vertebrate species present in the study area—for example, dogs, sheep, donkeys/horses, and a range of peri-domestic or wild mammals (rodents, small carnivores) were not tested and could account for a proportion of the unidentified meals. Second, blood meal degradation (due to the time between mosquito capture and sample preservation or suboptimal storage/transport conditions) can reduce the sensitivity of immunoassays and produce indeterminate results. Third, mixed blood meals may cause weak or ambiguous reactivity in ELISA-based assays, and cross-reactivity between closely related species can mask true host identity. Finally, the technical limitations of immunoassays (limits of detection, lot variability in antisera) can also increase the “unknown” fraction.

Because the unknown category is non-random, the observed host-feeding proportions likely underrepresent certain hosts and therefore should be interpreted cautiously. Conclusions about the relative importance of humans versus domestic animals as blood meal sources may be biased if a sizeable portion of anthropophagy or zoophagy is hidden within the unknowns. In particular, vector control recommendations that assume a low level of feeding on certain domestic animals should be made tentatively if these animals were not included in the assay panel.

## Figures and Tables

**Table 1:** The vertebrate host survey on the study site.

Hosts	Number	Percentage
Cattle	3933	20.1
Goat	163	0.9
Sheep	2184	11.2
Dog	520	2.7
Chicken	7159	36.7
Equine	496	2.5
Human	5065	25.9
Total	19,520	100

**Table 2:** Abdominal status of different Anopheles mosquito species collected from study area.

_Human Landing Catches (HLCs)	CDC Light Trap (CDC-LT)								Pyrethrum (PSC)								Pit Shelter (PS)								
	Outdoors				Indoors				Indoors				Outdoors				Indoor				Outdoor				Total
	UF	FF	HG	G	UF	FF	HG	G	UF	FF	HG	G	UF	FF	HG	G	UF	FF	HG	G	UF	FF	HG	G	
*An. coustani*	187	-	-	-	168	-	-	-	21	38	-	-	-	22	-	-	110	119	-	-	46	63	-	-	774
*An. gambiae* s.l.	432	185	-	-	426	-	-	-	225	150	2	21	130	116	3	10	232	978	-	-	26	46	-	-	2982
*An. cinereus*	164	-	-	-	83	-	-	-	37	18	13	5	20	21	1	32	18	74	-	-	-	74	1	-	561
*An. funestus*	35	-	-	-	25	-	-	-	-	13	-	-	1	18	1	3	19	16	1	7	16	13	1	7	176
*An. pharoensis*	146	-	-	-	115	-	-	-	7	16	7	4	18	17	1	30	71	110	1	-	14	19	1	28	605
Total	964	185	-	-	817	-	-	-	290	235	22	30	169	194	6	75	450	1297	2	7	102	215	3	35	5098

PSC = pyrethrum spray sheet collection; HLC = human landing catch outside and inside; UF = unfed; FF = freshly fed; HF = half-gravid; G = gravid.

**Table 3:** Allele-specific PCR (AS-PCR) for kdr genotyping.

_Genotype	Allele Frequency							
Species	RS	RR	SS	NB	Total	% Resistance (RS&RR)	% Susceptible (SS)	% Unidentified (NB)
*An. arabiensis*	3	38	171	39	251	16.3	68.13	15.54

RS = heterozygous resistant; RR = homozygous resistant; SS= homozygous susceptible; NB = No Band.

**Table 4:** Blood meal sources identified for *An. gambiae* s.l. collected from Jabi Tehnan District, Northwest Ethiopia (September 2016 to August 2017).

Collection Method	Collection Location	Bovine Blood Meal		Human Blood Meal		Mixed Blood Meal		Unidentified		Total
		no.	BBI (%)	no.	HBI (%)	no.	(%)	no.	(%)	no.
CDC-LT	Indoor	4	5.7	6	8.5	2	2.9	58	82.9	70
	Outdoor	3	6	2	4	2	4	43	86	50
HLC	Indoor	10	17.2	6	10.3	2	3.4	50	86.2	58
	Outdoor	2	6.9	10	34.5	5	17.2	12	41.4	29
PSC	Indoor	12	9.2	23	17.7	8	6.2	87	66.9	130
PS	Outdoor	3	13	6.	26.1	4	17.4	10	43.5	23
	Total	32	8.9	47	13.1	21	5.8	260	72.2	360

BBI = bovine blood index; HBI = human blood index; Mixed = human and bovine blood meal. Here, the abbreviations of the collection methods are also defined, i.e., PS = pit shelter; HLC = human landing catch; CDC = Centers for Disease Control; LT = Light Trap; PSC = Pyrethrum Spray Catch.

## Data Availability

The raw data supporting the conclusions of this article will be made available by the authors on request.

## References

[1] Coetzee M. (2020). Key to the females of Afrotropical Anopheles mosquitoes (*Diptera: Culicidae*). Malar. J..

[2] Coetzee M., Craig M., Le Sueur D. (2000). Distribution of African malaria mosquitoes belonging to the *Anopheles gambiae* complex. Parasitol. Today.

[3] Kweka E.J., Mazigo H.D., Lyaruu L.J., Mausa E.A., Venter N., Mahande A.M., Coetzee M. (2020). Anopheline mosquito species composition, kdr mutation frequency, and parasite infectivity status in northern Tanzania. J. Med. Entomol..

[4] Mustapha A.M., Musembi S., Nyamache A.K., Machani M.G., Kosgei J., Wamuyu L., Ochomo E., Lobo N.F. (2021). Secondary malaria vectors in western Kenya include novel species with unexpectedly high densities and parasite infection rates. Parasit. Vectors.

[5] Sinka M.E., Bangs M.J., Manguin S., Rubio-Palis Y., Chareonviriyaphap T., Coetzee M., Mbogo C.M., Hemingway J., Patil A.P., Temperley W.H. (2012). A global map of dominant malaria vectors. Parasit. Vectors.

[6] Hinne I.A., Attah S.K., Mensah B.A., Forson A.O., Afrane Y.A. (2021). Larval habitat diversity and Anopheles mosquito species distribution in different ecological zones in Ghana. Parasit. Vectors.

[7] Mireji P.O., Keating J., Hassanali A., Impoinvil D.E., Mbogo C.M., Muturi M.N., Nyambaka H., Kenya E.U., Githure J.I., Beier J.C. (2010). Expression of metallothionein and alpha-tubulin in heavy metal-tolerant *Anopheles gambiae* sensu stricto (*Diptera: Culicidae*). Ecotoxicol. Environ. Saf..

[8] Mireji P.O., Keating J., Hassanali A., Mbogo C.M., Nyambaka H., Kahindi S., Beier J.C. (2008). Heavy metals in mosquito larval habitats in urban Kisumu and Malindi, Kenya, and their impact. Ecotoxicol. Environ. Saf..

[9] Dahan-Moss Y., Hendershot A., Dhoogra M., Julius H., Zawada J., Kaiser M., Lobo N.F., Brooke B.D., Koekemoer L.L. (2020). Member species of the *Anopheles gambiae* complex can be misidentified as Anopheles leesoni. Malar. J..

[10] Gillies M.T., Coetzee M. (1987). A supplement to the Anophelinae of Africa South of the Sahara. Publ. S. Afr. Inst. Med. Res..

[11] Koekemoer L., Kamau L., Hunt R., Coetzee M. (2002). A cocktail polymerase chain reaction assay to identify members of the Anopheles funestus (*Diptera: Culicidae*) group. Am. J. Trop. Med. Hyg..

[12] Scott J.A., Brogdon W.G., Collins F.H. (1993). Identification of single specimens of the *Anopheles gambiae* complex by the polymerase chain reaction. Am. J. Trop. Med. Hyg..

[13] Wilkins E.E., Howell P.I., Benedict M.Q. (2006). IMP PCR primers detect single nucleotide polymorphisms for *Anopheles gambiae* species identification, Mopti and Savanna rDNA types, and resistance to dieldrin in Anopheles arabiensis. Malar. J..

[14] Muriu S.M., Muturi E.J., Shililu J.I., Mbogo C.M., Mwangangi J.M., Jacob B.G., Irungu L.W., Mukabana R.W., Githure J.I., Novak R.J. (2008). Host choice and multiple blood feeding behaviour of malaria vectors and other anophelines in Mwea rice scheme, Kenya. Malar. J..

[15] Bouafou L., Makanga B.K., Rahola N., Boddé M., Ngangué M.F., Daron J., Berger A., Mouillaud T., Makunin A., Korlević P. (2024). Host preference patterns in domestic and wild settings: Insights into Anopheles feeding behavior. Evol. Appl..

[16] Lefèvre T., Gouagna L.-C., Dabiré K.R., Elguero E., Fontenille D., Renaud F., Costantini C., Thomas F. (2009). Beyond nature and nurture: Phenotypic plasticity in blood-feeding behavior of *Anopheles gambiae* ss when humans are not readily accessible. Am. J. Trop. Med. Hyg..

[17] Mahande A., Mosha F., Mahande J., Kweka E. (2007). Feeding and resting behaviour of malaria vector, Anopheles arabiensis with reference to zooprophylaxis. Malar. J..

[18] Mahande A.M., Mosha F.W., Mahande J.M., Kweka E.J. (2007). Role of cattle treated with deltamethrine in areas with a high population of Anopheles arabiensis in Moshi, Northern Tanzania. Malar. J..

[19] Takken W., Lindsay S.W. (2003). Factors affecting the vectorial competence of Anopheles gambiae: A question of scale. Ecological Aspects for Application of Genetically Modified Mosquitoes.

[20] Mbewe R.B., Keven J.B., Mzilahowa T., Mathanga D., Wilson M., Cohee L., Laufer M.K., Walker E.D. (2022). Blood-feeding patterns of Anopheles vectors of human malaria in Malawi: Implications for malaria transmission and effectiveness of LLIN interventions. Malar. J..

[21] Orsborne J., Furuya-Kanamori L., Jeffries C.L., Kristan M., Mohammed A.R., Afrane Y.A., O’Reilly K., Massad E., Drakeley C., Walker T. (2018). Using the human blood index to investigate host biting plasticity: A systematic review and meta-regression of the three major African malaria vectors. Malar. J..

[22] Koffi A.A., Camara S., Ahoua Alou L.P., Oumbouke W.A., Wolie R.Z., Tia I.Z., Sternberg E.D., Yapo F.H., Koffi F.M., Assi S.B. (2023). Anopheles vector distribution and malaria transmission dynamics in Gbêkê region, central Côte d’Ivoire. Malar. J..

[23] Bashar K., Tuno N., Ahmed T.U., Howlader A.J. (2013). False positivity of circumsporozoite protein (CSP)–ELISA in zoophilic anophelines in Bangladesh. Acta Trop..

[24] Balkew M., Ibrahim M., Koekemoer L.L., Brooke B.D., Engers H., Aseffa A., Gebre-Michael T., Elhassen I. (2010). Insecticide resistance in Anopheles arabiensis (*Diptera: Culicidae*) from villages in central, northern and south west Ethiopia and detection of kdr mutation. Parasit. Vectors.

[25] Yewhalaw D., Wassie F., Steurbaut W., Spanoghe P., Van Bortel W., Denis L., Tessema D.A., Getachew Y., Coosemans M., Duchateau L. (2011). Multiple insecticide resistance: An impediment to insecticide-based malaria vector control program. PLoS ONE.

[26] Messenger L.A., Shililu J., Irish S.R., Anshebo G.Y., Tesfaye A.G., Ye-Ebiyo Y., Chibsa S., Dengela D., Dissanayake G., Kebede E. (2017). Insecticide resistance in Anopheles arabiensis from Ethiopia (2012–2016): A nationwide study for insecticide resistance monitoring. Malar. J..

[27] Liu N. (2015). Insecticide resistance in mosquitoes: Impact, mechanisms, and research directions. Annu. Rev. Entomol..

[28] Matowo J., Kulkarni M.A., Mosha F.W., Oxborough R.M., Kitau J.A., Tenu F., Rowland M. (2010). Biochemical basis of permethrin resistance in Anopheles arabiensis from Lower Moshi, north-eastern Tanzania. Malar. J..

[29] Wanjala C.L., Kweka E.J. (2018). Malaria vectors insecticides resistance in different agroecosystems in Western Kenya. Front. Public Health.

[30] Yewhalaw D., Kweka E.J. (2016). Insecticide resistance in East Africa—History, distribution and drawbacks on malaria vectors and disease control. Insectic. Resist..

[31] Dang K., Doggett S.L., Veera Singham G., Lee C.-Y. (2017). Insecticide resistance and resistance mechanisms in bed bugs, Cimex spp.(Hemiptera: Cimicidae). Parasit. Vectors.

[32] Messenger L.A., Impoinvil L.M., Derilus D., Yewhalaw D., Irish S., Lenhart A. (2021). A whole transcriptomic approach provides novel insights into the molecular basis of organophosphate and pyrethroid resistance in Anopheles arabiensis from Ethiopia. Insect Biochem. Mol. Biol..

[33] WHO (2023). World Malaria Report 2023.

[34] Kabula B., Tungu P., Rippon E.J., Steen K., Kisinza W., Magesa S., Mosha F., Donnelly M.J. (2016). A significant association between deltamethrin resistance, Plasmodium falciparum infection and the Vgsc-1014S resistance mutation in *Anopheles gambiae* highlights the epidemiological importance of resistance markers. Malar. J..

[35] Chanyalew T., Natea G., Amenu D., Yewhalaw D., Simma E.A. (2022). Composition of mosquito fauna and insecticide resistance status of *Anopheles gambiae* sensu lato in Itang special district, Gambella, Southwestern Ethiopia. Malar. J..

[36] Animut A., Negash Y., Kebede N. (2014). Distribution and utilization of vector control strategies in a malarious village of Jabi Tehnan District, north-western Ethiopia. Malar. J..

[37] Ayalew H., Dessalegn T., Liu H., Yan G. (2016). Performance of Ethiopian bread wheat (*Tritium aestivum* L.) genotypes under contrasting water regimes: Potential sources of variability for drought resistance breeding. Aust. J. Crop Sci..

[38] CSA (2013). Inter-Censal Population Survey Report.

[39] Tibebu T. (2016). Evaluation The Impact of World Vision Ethiopia, Water, Sanitation and Hygiene Project on The Community: The Case of Amhara Region, West Gojam Zone, Jabi Tehnane Woreda. Master’s Thesis.

[40] Wondie M., Alemneh T. (2018). The Prevalence of Bovine Trypanosomiasis in JabiTehnan District of Amhara Regional State, Ethiopia. Int. J. Cell Sci. Mol. Biol..

[41] WHO (1975). Manual on Practical Entomology in Malaria.

[42] Reddy M.R., Overgaard H.J., Abaga S., Reddy V.P., Caccone A., Kiszewski A.E., Slotman M.A. (2011). Outdoor host seeking behaviour of *Anopheles gambiae* mosquitoes following initiation of malaria vector control on Bioko Island, Equatorial Guinea. Malar. J..

[43] Geissbühler Y., Chaki P., Emidi B., Govella N.J., Shirima R., Mayagaya V., Mtasiwa D., Mshinda H., Fillinger U., Lindsay S.W. (2007). Interdependence of domestic malaria prevention measures and mosquito-human interactions in urban Dar es Salaam, Tanzania. Malar. J..

[44] Martinez-Torres D., Chandre F., Williamson M.S., Darriet F., Berge J.B., Devonshire A.L., Guillet P., Pasteur N., Pauron D. (1998). Molecular characterization of pyrethroid knockdown resistance (kdr) in the major malaria vector *Anopheles gambiae* s.s.. Insect Mol. Biol..

[45] Fanello C., Santolamazza F., Della Torre A. (2002). Simultaneous identification of species and molecular forms of the *Anopheles gambiae* complex by PCR-RFLP. Med. Vet. Entomol..

[46] Beier J.C., Asiago C.M., Onyango F.K., Koros J.K. (1988). ELISA absorbance cut-off method affects malaria sporozoite rate determination in wild Afrotropical Anopheles. Med. Vet. Entomol..

[47] Wirtz R.A., Sattabongkot J., Hall T., Burkot T.R., Rosenberg R. (1992). Development and evaluation of an enzyme-linked immunosorbent assay for Plasmodium vivax-VK247 sporozoites. J. Med. Entomol..

[48] Shililu, Maier, Seitz, Orago (1998). Seasonal density, sporozoite rates and entomological inoculation rates of Anopheles gambiae and Anopheles funestus in a high-altitude sugarcane growing zone in western kenya. Trop. Med. Int. Health.

[49] Rosenberg R., Wirtz R.A., Schneider I., Burge R. (1990). An estimation of the number of malaria sporozoites ejected by a feeding mosquito. Trans. R. Soc. Trop. Med. Hyg..

[50] Blanken S.L., O’Meara W.P., Hol F.J., Bousema T., Markwalter C.F. (2024). À la carte: How mosquitoes choose their blood meals. Trends Parasitol..

[51] Fikrig K., Harrington L.C. (2021). Understanding and interpreting mosquito blood feeding studies: The case of Aedes albopictus. Trends Parasitol..

[52] Shaw W.R., Holmdahl I.E., Itoe M.A., Werling K., Marquette M., Paton D.G., Singh N., Buckee C.O., Childs L.M., Catteruccia F. (2020). Multiple blood feeding in mosquitoes shortens the Plasmodium falciparum incubation period and increases malaria transmission potential. PLoS Pathog..

[53] Ashine T., Eyasu A., Asmamaw Y., Simma E., Zemene E., Epstein A., Brown R., Negash N., Kochora A., Reynolds A.M. (2024). Spatiotemporal distribution of *Anopheles stephensi* in different eco-epidemiological settings in Ethiopia. Parasit Vectors.

[54] Charlwood J., Kessy E., Yohannes K., Protopopoff N., Rowland M., LeClair C. (2018). Studies on the resting behaviour and host choice of *Anopheles gambiae* and An. arabiensis from Muleba, Tanzania. Med. Vet. Entomol..

[55] Assogba B.S., Pasteur N., Makoundou P., Unal S., Baba-Moussa L., Labbé P., Weill M. (2020). Dynamic of resistance alleles of two major insecticide targets in Anopheles gambiae (s.l.) populations from Benin, West Africa. Parasit Vectors.

[56] Adugna T., Getu E., Yewhalaw D. (2020). Species diversity and distribution of Anopheles mosquitoes in Bure district, Northwestern Ethiopia. Heliyon.

[57] Hailemariam A., Dugassa S., Kweka E., Tekie H. (2022). Population dynamics, biting activities and resting behavior of Anopheles mosquitoes in Jabi Tehnan district, Northwest Ethiopia: Implications for indoor malaria vector control. Ethiop. J. Public Health Nutr. (EJPHN).

[58] Kenea O., Balkew M., Tekie H., Deressa W., Loha E., Lindtjørn B., Overgaard H.J. (2019). Impact of combining indoor residual spraying and long-lasting insecticidal nets on Anopheles arabiensis in Ethiopia: Results from a cluster randomized controlled trial. Malar. J..

[59] WHO (2018). Global Report on Insecticide Resistance in Malaria Vectors: 2010–2016.

[60] Karunaratne S., De Silva W., Weeraratne T.C., Surendran S.N. (2018). Insecticide resistance in mosquitoes: Development, mechanisms and monitoring. Ceylon J. Sci..

[61] Abdu H.U. (2015). Investigating the Role of Glutathione and Glutathione Biosynthetic Genes in the Adaptation of *Anopheles gambiae* to Insecticides. Ph.D. Thesis.

[62] Asale A., Duchateau L., Devleesschauwer B., Huisman G., Yewhalaw D. (2017). Zooprophylaxis as a control strategy for malaria caused by the vector Anopheles arabiensis (*Diptera: Culicidae*): A systematic review. Infect. Dis. Poverty.

[63] Taye B., Lelisa K., Emana D., Asale A., Yewhalaw D. (2016). Seasonal Dynamics, Longevity, and Biting Activity of Anopheline Mosquitoes in Southwestern Ethiopia. J. Insect Sci..

[64] Matowo J., Kitau J., Kaaya R., Kavishe R., Wright A., Kisinza W., Kleinschmidt I., Mosha F., Rowland M., Protopopoff N. (2015). Trends in the selection of insecticide resistance in *Anopheles gambiae* sl mosquitoes in northwest T anzania during a community randomized trial of longlasting insecticidal nets and indoor residual spraying. Med. Vet. Entomol..

[65] Ochomo E.O., Bayoh N.M., Walker E.D., Abongo B.O., Ombok M.O., Ouma C., Githeko A.K., Vulule J., Yan G., Gimnig J.E. (2013). The efficacy of long-lasting nets with declining physical integrity may be compromised in areas with high levels of pyrethroid resistance. Malar. J..

[66] Abebe W., Sisay A., Mihret Y., Setegn A., Asmare Z., Woldesenbet D., Kassanew B., Mekuanint A., Feleke S.F. (2025). Prevalence of *Anopheles stephensi* in Horn of Africa: A systematic review and meta-analysis. BMC Infect. Dis..

[67] Carter T.E., Yared S., Gebresilassie A., Bonnell V., Damodaran L., Lopez K., Ibrahim M., Mohammed S., Janies D. (2018). First detection of *Anopheles stephensi* Liston, 1901 (*Diptera: Culicidae*) in Ethiopia using molecular and morphological approaches. Acta Trop..

[68] Degefa T., Zhong D., Lee M.C., Merga H., Abiy E., Wang X., Zhou G., Kifle T., Yewhalaw D., Yan G. (2025). Bionomics of *Anopheles stephensi* across the urban-rural landscapes of Eastern Ethiopia. Malar. J..

[69] Seyfarth M., Khaireh B.A., Abdi A.A., Bouh S.M., Faulde M.K. (2019). Five years following first detection of *Anopheles stephensi* (*Diptera: Culicidae*) in Djibouti, Horn of Africa: Populations established-malaria emerging. Parasitol. Res..

